# Dissipation and Residues of Pyrethrins in Leaf Lettuce under Greenhouse and Open Field Conditions

**DOI:** 10.3390/ijerph14070822

**Published:** 2017-07-21

**Authors:** Lixiang Pan, Xiaoxiao Feng, Hongyan Zhang

**Affiliations:** College of Science, China Agricultural University, Beijing 100193, China; panlixiang5545@cau.edu.cn (L.P.); xxf0423@cau.edu.cn (X.F.)

**Keywords:** dissipation, residue, leaf lettuce, pyrethrins, GC/MS

## Abstract

Pyrethrins are nowadays widely used for prevention and control of insects in leaf lettuce. However, there is a concern about the pesticide residue in leaf lettuce. A reliable analytical method for determination of pyrethrins (pyrethrin—and П, cinerin І and П, and jasmolin І and П) in leaf lettuce was developed by using gas chromatography–mass spectrometry (GC–MS). Recoveries of pyrethrins in leaf lettuce at three spiking levels were 99.4–104.0% with relative standard deviations of 0.9–3.1% (*n* = 5). Evaluation of dissipation and final residues of pyrethrins in leaf lettuce were determined at six different locations, including the open field, as well as under greenhouse conditions. The initial concentration of pyrethrins in greenhouse (0.57 mg/kg) was higher than in open field (0.25 mg/kg) and the half-life for pyrethrins disappearance in field lettuce (0.7 days) was less than that greenhouse lettuce (1.1 days). Factors such as rainfall, solar radiation, wind speed, and crop growth rate are likely to have caused these results. The final residue in leaf lettuce was far below the maximum residue limits (MRLs) (1 mg/kg established by the European Union (EU), Australia, Korea, Japan).

## 1. Introduction

Leaf lettuce belongs to *Lactuca compositae* and originated on the Mediterranean coast. Leaf lettuce is one kind of high nutrition vegetable, which contains vitamins (A, B_1_, B_2_), calcium, iron and other nutrients [1]. It is a low-calorie vegetable, which, amongst other benefits, can lower cholesterol, treat neurasthenia and clear the lungs [2].

**Figure 1:**
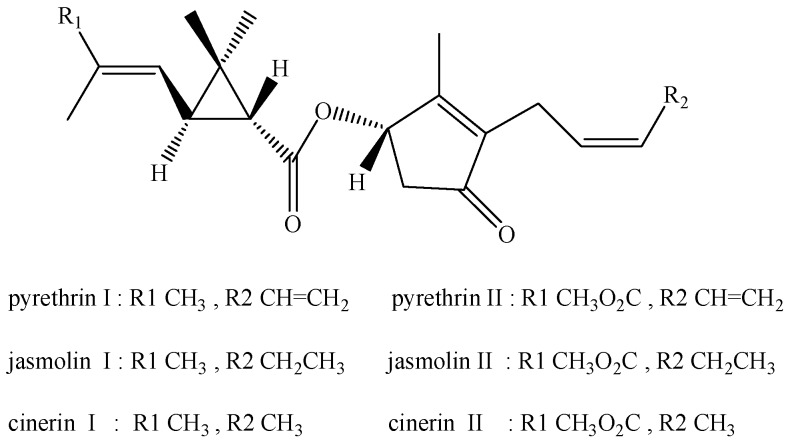
The chemical structures of pyrethrins.

Pyrethrins, which have the function of causing paralysis to an insect’s central nervous system, are used as neurotoxic agents to control aphids on leaf lettuce [3]. Pyrethrins are naturally occurring compounds extracted from the powdered flower-heads of *Chrysanthemum cinerariaefolium*. The term “pyrethrum” and “pyrethrins” are easily confused, with the term “pyrethrins” being reserved for describing the active insecticidal components of “pyrethrum” [4]. Pyrethrins are widely used in the world due to their excellent characteristics, including being broad-spectrum, highly efficient, low in toxicity, non-persistent, do not easily produce drug resistance, and environmentally friendly [5]. Pyrethrins have six insecticidal active compounds including three esters of chrysanthemum acid (cinerin I, jasmolin I, pyrethrin I-called pyrethrins I) and three esters of pyrethrum acid (cinerin II, jasmolin II, pyrethrin II-called pyrethrins II). The chemical structures of pyrethrins are shown in Figure 1 [3]. Pyrethrin I and pyrethrin II are the main ingredients. Pyrethrins I have a strong lethal activity against insects, while pyrethrins II have a strong knockdown activity [6].

The Joint Meeting on Pesticide Residues (JMPR) reported that the pyrethrins residue definition for compliance with maximum residue limits (MRLs) and estimation of dietary intake of total pyrethrins, is calculated as the sum of six insecticidal active compounds pyrethrin I, pyrethrin II, cinerin I, cinerin II, jasmolin I and jasmolin II (http://www.fao.org/fileadmin/templates/agphome/documents/Pests_Pesticides/JMPR/Reports 1991-2006/Report 2000.pdf). Simultaneous separation of the six components is very difficult because of their similar structures. Natural pyrethrins always appear together, and the cost of single chemical component analysis is higher [7]. Therefore, a reliable, specific and sensitive analytical method for the sum of pyrethrins is required.

In the past few years, many analytical methods for the determination of pyrethrins and their dissipation in some matrices have been reported. A quick, easy, cheap, effective, rugged, and safe (QuEChERS) methodology for the determination of pyrethrins in lemon and apricot has been developed using high-performance liquid chromatography-mass spectrometry (HPLC-MS) [8], in fish tissues by gas chromatography–mass spectrometry (GC–MS) [9], and in foods by ultra-performance liquid chromatography-mass spectrometry (UPLC-MS) [10]. There are also studies to detect the residues of pyrethrins in tea samples by ultra-performance liquid chromatography coupled with tandem mass spectrometry (UPLC-MS/MS) [11] in surface water by high-resolution gas chromatography/high-resolution mass spectrometry (HRGC/HRMS) [12], and in fruits and vegetables by LC-MS/MS [6] with solid phase extraction (SPE) cartridges or no clean-up. Antonious et al. [13] reported the half-life (T_1/2_) values of pyrethrins on pepper and tomato fruits did not exceed 2 h, while Angioni et al. [14] reported the half life of total pyrethrins on peaches within 2.3 days after field treatments.

The use of the greenhouse will continue to grow globally, as greenhouses are becoming increasingly important in the supply of fresh fruits and vegetables. However, studies on the environmental behavior of pesticides in greenhouses are very limited [15]. Therefore, it is relevant to investigate the dissipation and residues of pyrethrins in leaf lettuce under both open field and greenhouse conditions. Moreover, the maximum residue limit (MRL) regulations require a preharvest interval (PHI) to ensure that the dissipation of a pesticide is below the proposed MRL at harvest [16]. The aim of this project was establish a residue analysis method to investigate the dissipation trend and final residue levels of pyrethrins in leaf lettuce under open field and greenhouse conditions. These results will provide guidance on the proper and safe use of pyrethrins.

## 2. Materials and Methods

### 2.1. Materials and Reagents

Standard Pyrethrin II (*w*/*w*, 30%, technical Grade, contains Pyrethrin I) was obtained from Toronto Research Chemicals Inc. (Toronto, ON, Canada). Sodium chloride and anhydrous sodium sulfate were analytical reagent grade and purchased from Beijing Chemical Reagent Company (Beijing, China). Acetonitrile, acetone and *n*-hexane were both HPLC-grade and purchased from Fisher Scientific (Pittsburgh, PA, USA). Aquapro Ultrapure Water System supply ultrapure water. Florisil SPE cartridges (0.5 g/3 mL) were purchased from ANPEL (Shanghai, China). Stock standard solution of pyrethrins (1000 mg/L) was prepared in acetone and stored at −20 °C. Working standard solutions were prepared by dilution with acetone and stored at −20 °C.

### 2.2. Field Trials

The field trials, including the dissipation and residue experiments, were carried out in six different locations: Daxing district (Beijing, north China, warm and semihumid continental monsoon climate); Jinan (Shandong Province, north China, temperate continental monsoon climate); Jurong (Jiangsu Province, east China, subtropical monsoon climate); Hangzhou (Zhejiang Province, east China, subtropical monsoon climate); Yueyang (Hunan Province, middle of China, continental subtropical monsoon humid climate); and Haikou (Hainan Province, south China, tropical monsoon climate), in 2016, according to the Guidelines for Pesticide Residue Field Experiments (NY/T 788–2004), published by the Ministry of Agriculture, People’s Republic of China. The designs of the field trials are shown in Table 1. There were six treatments, including five pyrethrins treatments and one control treatment. Each treatment consisted of three replicate plots and each experimental plot was 15 m^2^. In the control treatment, the whole growth period of leaf lettuce was carried out with no pesticide used. The plots of different treatments used a buffer area (30 m^2^) to separate.

In order to investigate the dissipation dynamics of pyrethrins in leaf lettuce, formulations of pyrethrins (emulsion in water (EW), 1.5%) were dissolved in water and sprayed with an active constituent dose of 54 g a.i./ha (1.5 times the recommended high dosage) on the leaf lettuce. It was applied once in the middle of leaf lettuce growth. Representative leaf lettuce was randomly collected from each plot at 2 h, 1, 2, 3, 5, 7, 10, 14, 21 days after spraying.

### 2.3. Sampling and Storage

Leaf lettuce samples for dissipation and terminal residue experiments were randomly collected from each plot at different time intervals after treatment. About 1000 g leaf lettuce samples were randomly collected in each plot. The leaf lettuce samples were cut into small pieces and homogenized with a blender at 12,000 rpm for 1 min (Philips, HR2100, Shanghai, China). Sub-sampling of 200 g leaf lettuce was put in the plastic boxes or bags. All samples were kept in a deep freezer at −18 °C for analysis within 2 months.

### 2.4. Sample Preparation

A portion (20.0 g) of blended leaf lettuce was weighed into a 50 mL centrifuge tube, and then 20 mL acetonitrile and 3 g sodium chloride were added. The samples were extracted by shaking on an oscillator at 140 rpm for 30 min and then centrifuged at 3800 rpm for 5 min. Ten mL of the supernatant were transferred into 100 mL chicken heart bottle, vacuum evaporated to dryness, and dissolved in 1 mL of acetone/*n*-hexane (*v*/*v*, 1/9) for cleanup.

Florisil SPE cartridge was activated with 10 mL *n*-hexane after adding 0.5 cm of anhydrous sodium sulfate. One mL concentrated sample was added to the top of Florisil SPE cartridge and eluted with 10 mL of acetone/*n*-hexane (*v*/*v*, 1/9) at the speed of 2 mL/min. The eluate was collected with chicken heart bottle and vacuum evaporated to dryness, dissolved in 1 mL of acetone, and analyzed by GC–MS after filtered with 0.22 µm syringe filters.

### 2.5. GC–MS Condition

**Figure 2:**
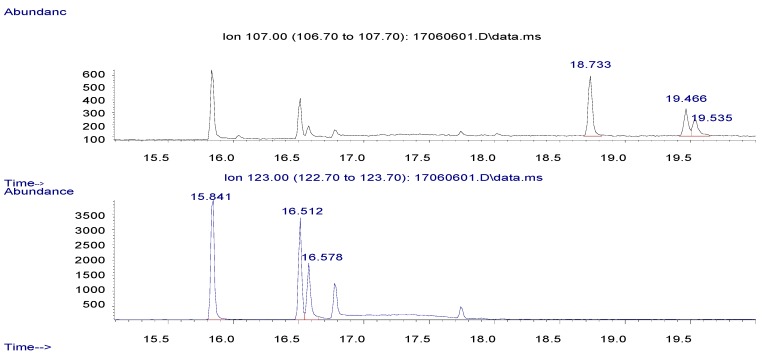
The gas chromatography–mass spectrometry (GC–MS) chromatograph of pyrethrins (10 mg/L).

Pyrethrins were analyzed by Agilent 6890N Network GC system equipped with an Agilent 7683 series auto sampler, mass selective detector (MSD) model 5975B network, and a HP-5MS analytical column (30 m × 0.25 mm × 0.25 μm film thickness) (Agilent Technologies, Santa Clara, CA, USA). Two μL of sample were injected in splitless mode, and the injector was held at 260 °C, the carrier gas helium (99.999%) at a constant flow rate of 1.2 mL/min. The oven temperature was initially held at 100 °C for 1 min, ramped to 270 °C at 10 °C/min and held for 1 min, ramped to 280 °C at 1 °C/min and kept for 1 min. The temperatures of ion source, quadrupole, and transfer line were set as 230 °C, 150 °C, and 280 °C. The solvent delay was 10 min. Ionization was performed using electron impact (EI) mode at 70 eV, and mass spectrometer was used in selected ion monitoring (SIM) mode. The total running time was 30 min. Table 2 summarizes the ions monitored along with the relative abundances and the typical retention time. The chromatograph is shown in Figure 2.

### 2.6. Statistical Analysis

The dissipation pattern of pyrethrins in leaf lettuce was fitted to the first-order kinetics Equation (1):

*C* = *C*_0_*e*^-*kt*^ (1)

The *DT*_50_ of pyrehrins was calculated separately for each location using Hoskins’ Formula (2) [17]:

*DT*_50_ = (ln 2)/*k* (2)

where *C* is the concentration of pyrethrins (mg/kg) at time *t* (day), *C*_0_ is the initial concentration of pyrethrins (mg/kg), and *k* is the first-order rate constant (*d* − 1) independent of *C* and *C*_0_. *DT*_50_ is the time to reach half of the initial residual level of pyrethrins after application.

## 3. Results and Discussion

### 3.1. Optimization of GC–MS Conditions

In this study, the results of selectivity and peak shape can be achieved by choosing a type of HP-5 MS as the optimal column. The monitoring ions of pyrethrins referenced determination of pyrethrins residuses in cereals for export-GC–MS method (SN/T 0218-2014), and the optimum detection parameters were determined in SIM mode. The solvent delay was 10 min to protect the ion source. Temperature programming plays an important role in adjusting retention time, selectivity, and peak shape as they are important parameters in GC separation [18]. The initial temperature was ramped to 270 °C at 5, 10, 15, 20 °C/min, and 10 °C/min gave a better peak shape. During the 270 °C raised to 280 °C, jasmolin II and pyrethrin II appeared. When 270 °C was ramped to 280 °C at 0.5, 1, 2 °C/min, then 1 °C/min gave a better peak shape and better separation.

### 3.2. Optimizations of Sample Pretreatment

An accurate and sensitive analysis method for pyrethrins in leaf lettuce was investigated in this study. Leaf lettuce is one kind of complex matrix which is relatively difficult for pyrethrins extraction. The matrix clean-up efficiency is an important factor for the analysis results. Acetonitrile was used as the extractant to ensure good extraction efficiencies for pyrethrins. Previous reports have shown that the QuEChERS method could be applied to extract pyrethrins in lemon and apricot [8]. Based on that, dispersive solid-phase extraction (d-SPE) was attempted. A total of 25, 50, 75, 100 mg PSA or C_18_ and 100 mg anhydrous magnesium sulfate (MgSO_4_) was chosen as the cleanup sorbent for the extraction of pyrethrins in leaf lettuce, while the recoveries of C18 were not satisfactory and 50 mg PSA had best satisfactory recovery. The limit of quantification (LOQ) value of pyrethrins in leaf lettuce was 0.5 mg/kg, by using 50 mg PSA and 100 mg MgSO_4_. However, the sensitivity of pyrethrins in leaf lettuce was unsatisfactory, due to the large matrix interference, meanwhile the chlorophyll was only slightly reduced by using PSA and could build up in the injection port liner and increase the frequency of liner changes and column maintenance [18].

**Figure 3:**
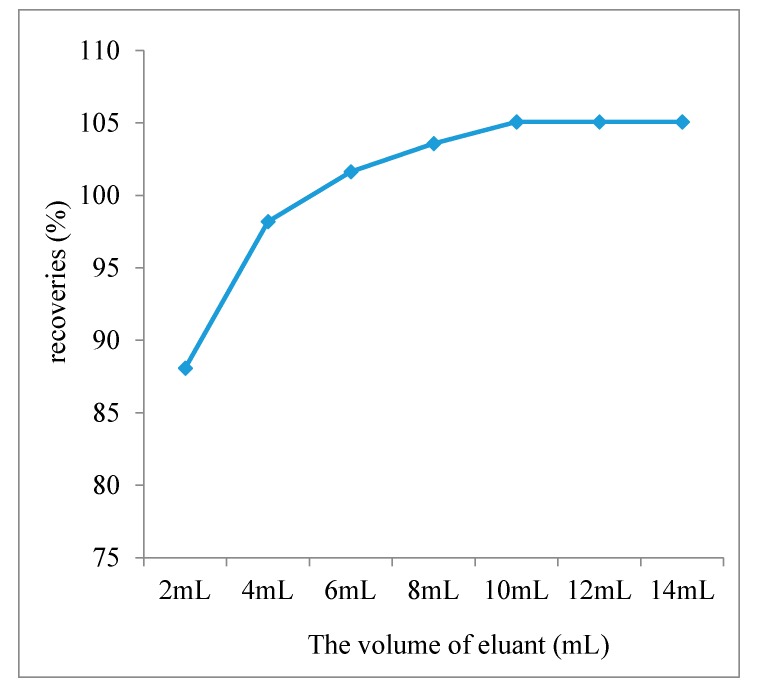
The elution curve of pyrethrins.

Then the clean-up procedure was optimized. SPE technique is a classical and reliable residue analytical method to purify matrix and samples concentration, in order to obtain a high sensitivity [11]. Although SPE cartridges are typically 2–3 times more expensive than purchasing the sorbents directly, the use of cartridges could have 100% of the extract volume that would be recovered during the cleanup step [18]. According to the reference of SN/T 0218-2014, Florisil SPE cartridge was activated with 10 mL *n*-hexane after adding 0.5 cm of anhydrous sodium sulfate. Acetone–*n*-hexane (*v*/*v*) 0:10, 1:9, 2:8 and 3:7 as the eluant were compared; the results showed acetone–*n*-hexane (*v*/*v*, 1:9) gave a higher response, better peak shape and less impurities. The volume of acetone–*n*-hexane (*v*/*v*, 1:9) was also optimized. The elution curve is shown in Figure 3. The results showed that 10 mL acetone–*n*-hexane (*v*/*v*, 1:9) as the eluant can make pyrethrins totally eluted from the SPE cartridges.

### 3.3. Method Validation

In order to evaluate the linearity and sensitivity of the analytical method for pyrethrins, a series of matrix standard solutions (0.2, 0.5, 1, 2, 5, 10 mg/L) were diluted by leaf lettuce blank matrix extract, each point was repeated three times. The standard calibration curve of the sum of pyrethrins was constructed by plotting the average peak area against concentration. The calibration curves showed good linearity with typical correlation coefficients (R^2^) between 0.9970 and 0.9996. It was used to calculate the real residue concentration of pyrethrins in leaf lettuce samples. The limit of detection (LOD) value was 4.0 × 10^−10^ g and the LOQ value was 0.05 mg/kg. To evaluate method accuracy and precision, three fortified levels for matrix with five duplicates was conducted in leaf lettuce. The results were listed in Table 3. The recovery and precision results were satisfactory according to the residue analysis quality control guide (General Administration of Quarantine of the People’s Republic of China, 2002). The matrix-matched standard calibrations method was used to eliminate the matrix effect. Matrix effects were calculated with the equation [19]:

$$\mathrm{Matrix}\;\mathrm{effect}\left(\%\right)\;=\;\left(\left(\frac{\mathrm{areas}\;\mathrm{in}\;\mathrm{matrix}}{\mathrm{areas}\;\mathrm{in}\;\mathrm{acetone}}\right)-1\right)\times 100$$

The calculated matrix effects for pyrethrins in leaf lettuce matrice was lower than 20% and can be viewed as insignificant [20].

### 3.4. Dissipation of Pyrethrins in Leaf Lettuce under Greenhouse and Open Field Conditions

**Figure 4:**
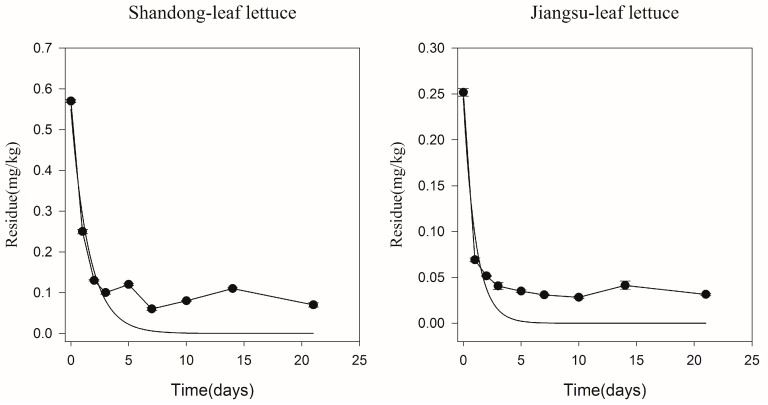
Dissipation of pyrethrins residue in leaf lettuce in Shandong and Jiangsu.

Two locations (greenhouse test in Shandong, open field test in Jiangsu) were chosen to investigate dissipation of pyrethrins in leaf lettuce. The dissipation curve of pyrethrins in leaf lettuce was fitted with first order kinetics as shown in Figure 4. The initial concentration of pyrethrins in the greenhouse (0.57 mg/kg) was higher than that in open field (0.25 mg/kg). This result agreed with Sharma’s report, in which initial residues of acephate were higher in capsicum grown in the greenhouse than that in the open field [21]. The dissipation equation of pyrethrins in leaf lettuce were *C* = 0.5498*e*^−0.6387*t*^ with correlation coefficient (R) of 0.9031 in the greenhouse and *C* = 0.2450*e*^−0.9312*t*^ with correlation coefficient (R) of 0.9085 in the open field. In a single experiment, the half-life of pyrethrins in leaf lettuce under open field conditions (0.7 days) was less than that for greenhouse conditions (1.1 days). Different initial concentrations and *DT*_50_ were affected by climate differences between greenhouse and open field during leaf lettuce growth. Initial concentration in the open field was less, probably due to volatilization and wind drift. The entire spray application settles down on the plant in the greenhouse, as there is no wind drift and very little volatilization losses during or immediately after the application, and the crop density is also higher, which might cause a higher initial concentration under greenhouse conditions [21]. Pesticides accumulation on the plant can significantly decrease under open field conditions, due to the direct effect of rainfall [22]. The annual precipitation of the bai-u rainy period in Jiangsu (open field) is above 1000 mm, while the influence of rainfall is considered to be negligible on the dissipation of pesticide residues in greenhouse conditions (Shandong) [23]. The factors such as solar radiation and crop growth rate also can cause different degradation rates [21].

### 3.5. Terminal Residue Analysis

The terminal residue samples were analyzed in six locations. The results of pyrethrins in leaf lettuce collected at harvest time are listed in Table 4. Under greenhouse conditions, the residues in pre-harvest intervals (PHIs) 2, 3, and 5 days’ leaf lettuce samples were 0.08–0.17 mg/kg in Shandong and 0.08–0.30 mg/kg in Zhejiang. The annual average sunshine duration of Zhejiang is 1710–2100 h, while Shandong is 2290–2890 h. Pyrethrins are rapidly degraded under sunlight. Under open field conditions, the highest residue of pyrethrins in Beijing (0.36 mg/kg) and Hunan (0.36 mg/kg), which were higher than the residue of pyrethrins in Jiangsu (0.08 mg/kg) and Hainan (0.11 mg/kg). During planting, the average daily temperatures of Jiangsu and Hainan were higher than Beijing and Hunan 5–10 °C, and pyrethrins degraded quickly at high temperatures, so there are lower residues in Jiangsu and Hainan. There was no difference at different intervals in the residue of Jiangsu and Hainan, and it might be because the residues in leaf lettuce were all very low (≤0.11 mg/kg). By comparing the greenhouse with open field conditions, the residues of pyrethrins showed no significant difference. This result was consistent with the report of Bojacá [22] that there was no indication about which farming system (open field and greenhouse) produced the most contaminated tomatoes. It showed when pyrethrins were used under the designed experiment, the terminal residue of pyrethrins in leaf lettuce was far below the MRLs (1 mg/kg established by the European Union (EU), Australia, Korea and Japan). The residue data suggests that the pyrethrins EW formulation can be safe applied in leaf lettuce at a dosage of 36–54 g a.i./ha with a PHI of 2 days.

## 4. Conclusions

A reliable modified analytical method with GC–MS was applied for the determination of pyrethrins in leaf lettuce. The method used for extraction, clean-up and estimation of pyrethrins in leaf lettuce was found to be satisfactory, qualitatively as well as quantitatively. Pyrethrins rapidly degraded in leaf lettuce. The initial concentration of pyrethrins in greenhouse (0.57 mg/kg) was higher than that in the open field (0.25 mg/kg) and the half-lives of pyrethrins in leaf lettuce ranged from 0.7 (greenhouse) to 1.1 (open field) days. By comparing greenhouse with open field conditions, the residues of pyrethrins showed no difference. The residue of pyrethrins is affected by factors such as light, temperature, humidity, rainfall, wind speed, and crop growth rate. Compared with the recommended MRLs for pyrethrins in leaf lettuce (1 mg/kg established by EU, Australia, Korea, Japan), the final residues of pyrethrins in leaf lettuce was much lower than 1 mg/kg when PHIs were set as 2, 3, and 5 days. Our findings suggest that pyrethrins of 1.5% EW could be safely used in leaf lettuce with the recommended dosage. This study could provide the data for the Chinese government to establish the MRL of pyrethrins in leaf lettuce, and also provide guidance on the proper and safe use of the pesticide in agricultural products and the environment.

## Figures and Tables

**Table 1:** Design of the field experiments for pyrethrins residue and dissipation in leaf lettuce.

Treatments		Dosage of Application ^c^	Times of Application	Experiments	Days after Treatment
Serial number ^a^	Area (m^2^)	g a.i./ha			
1	15 * 3 ^b^	36	2	Ultimate residue ^d^	2, 3, 5
2	15 * 3	36	3	Ultimate residue ^d^	2, 3, 5
3	15 * 3	54	2	Ultimate residue ^d^	2, 3, 5
4	15 * 3	54	3	Ultimate residue ^d^	2, 3, 5
5	15 * 3	54	1	Dissipation in leaf lettuce ^e^	2 h, 1, 2, 3, 5, 7, 10, 14, 21
6	30	0	-	Control	Before the spraying and harvest

Notes: ^a^: These serial numbers mean there were six treatments; ^b^: 15 * 3 means each experimental plot was 15 m^2^ and each treatment consisted of three replicate plots, * is multiplication sign; ^c^: 36 g a.i./ha is the recommended dosage and 54 g a.i./ha is 1.5 times of the recommended dosage, the re-treatment interval was 7 days; ^d^: Ultimate residue were experimented at six locations, including greenhouse (Shandong and Zhejiang) and open field (Beijing, Jiangsu, Hunan and Hainan); ^e^: Dissipation in leaf lettuce only experimented at Shandong (Greenhouse) and Jiangsu (Open field).

**Table 2:** Monitor ions with the relative abundances and the typical retention time of pyrethrins.

Compounds	Monitor Ions (*m*/*z*)	Relative Ion Abundance Ratio (%)	Relative Retention Time (min)
cnerin I	123 *, 150,168	100:23:3	15.84
jasmolin I	123 *, 135,69	100:12:4	16.51
pyrethrin I	123 *, 81,105,162	100:21:21:16	16.57
cinerin II	107 *, 121,93,167	100:79:53:53	18.73
jasmolin II	107 *, 93,121,167	100:63:55:54	19.47
pyrethrin II	107 *, 91,167	100:87:59	19.54

Note: * Quantitative ion.

**Table 3:** Recoveries and relative standard deviation (RSD) of the sum of pyrethrins in leaf lettuce

Matrix	Fortified Level (mg/kg)	Fortified Recovery (%)	RSD (%)
**leaf lettuce**	0.05	104.0 ± 3.3	3.1
	0.1	103.4 ± 1.0	0.9
	1	99.4 ± 2.8	2.9

**Table 4:** Final residues of pyrethrins in leaf lettuce samples.

Dosage (ga.i./ha)	36						54					
Numbers of times sprayed	2			3			2			3		
Pre-harvest intervals	2	3	5	2	3	5	2	3	5	2	3	5
Residue(mg/kg)												
Beijing (open field)	0.10	0.07	0.05	0.20	0.11	0.10	0.28	0.15	0.09	0.36	0.22	0.15
Jiangsu (open field)	<0.05	<0.05	<0.05	0.06	0.06	0.05	0.08	<0.05	<0.05	0.08	<0.05	<0.05
Hunan (open field)	0.22	0.19	0.11	0.18	0.13	0.14	0.36	0.19	0.14	0.22	0.19	0.14
Hainan (open field)	0.08	0.06	0.09	0.06	0.06	0.06	0.11	0.06	0.06	0.06	0.11	0.09
Shandong (greenhouse)	0.14	0.09	0.08	0.17	0.12	0.15	0.10	0.12	0.09	0.11	0.11	0.09
Zhejiang (greenhouse)	0.30	0.14	0.10	0.14	0.08	0.11	0.27	0.15	0.09	0.21	0.15	0.08
